# Wild-type but not mutant SOD1 transgenic astrocytes promote the efficient generation of motor neuron progenitors from mouse embryonic stem cells

**DOI:** 10.1186/1471-2202-14-126

**Published:** 2013-10-17

**Authors:** Yiota A Christou, Kyoji Ohyama, Marysia Placzek, Peter N Monk, Pamela J Shaw

**Affiliations:** 1 Department of Neuroscience, Sheffield Institute for Translational Neuroscience (SITraN), University of Sheffield, Sheffield, S10 2HQ, UK; 2 Department of Infection and Immunity, Faculty of Medicine, Dentistry and Health, University of Sheffield, Sheffield, S10 2HQ, UK; 3 MRC Centre for Developmental and Biomedical Genetics and Department of Biomedical Science, University of Sheffield, Sheffield, S10 2RX, UK

**Keywords:** ES cells, G93A astrocytes, Motor neuron, Progenitor, Differentiation, ALS

## Abstract

**Background:**

The efficient derivation of mature (Hb9+) motor neurons from embryonic stem cells is a sought-after goal in the understanding, and potential treatment, of motor neuron diseases. Conditions that promote the robust generation of motor neuron progenitors from embryonic stem cells and that promote the survival of differentiated motor neurons *ex vivo* are likely, therefore, to be critical in future biological/therapeutic/screening approaches. Previous studies have shown that astrocytes have a protective effect on differentiated motor neurons (*in vivo* and *ex vivo*), but it remains unclear whether astrocytes also play a beneficial role in the support of motor neuron progenitors. Here we explore the effect of murine astrocyte-conditioned medium on monolayer cultures of mouse embryonic stem cell-derived motor neuron progenitors.

**Results:**

Our data show that wild-type astrocyte-conditioned medium significantly increases the number of Olig2+/Hb9- progenitors, which subsequently differentiate into Hb9+/Islet1+ post-mitotic motor neurons. Intriguingly, while astrocyte-conditioned medium derived from mice transgenic for wild-type human *SOD1* mimics the effect of wild-type astrocytes, conditioned medium derived from astrocytes carrying an amyotrophic lateral sclerosis-related *SOD1*-*G93A* mutation shows no such beneficial effect. The effect of astrocyte-conditioned medium, moreover, is specific to motor neurons: we find that interneurons generated from mouse embryonic stem cells are unaffected by conditioned media from any type of astrocyte.

**Conclusions:**

Our study indicates that conditioned medium derived from wild type astrocytes enhances the efficient generation of motor neurons from mouse embryonic stem cells by enhancing motor neuron progenitors. In contrast, conditioned medium from *SOD1*-*G93A* astrocytes does not show a similar enhancing effect.

## Background

Motor neurons (MNs) are selectively depleted, or diminished, in motor neuron diseases such as amyotrophic lateral sclerosis (ALS). Various factors contribute to the pathogenesis of ALS including mutations in the *SOD1* gene, mutations in the *ALS2* (Alsin) [1,2], *FUS* (RNA-binding protein FUS) [3], *TARDBP* (TAR DNA-binding protein 43) [4,5], *ATXN2* (Ataxin-2) [6] and *ANG* (Angiogenin) [7] genes, and the recently-discovered intronic hexanucleotide expansions in *C9ORF72*[8,9]. In mutant SOD1 disease models, non-neuronal cells, such as astrocytes have been implicated in the pathogenesis of MN degeneration [10-12]. The mechanism by which astrocytes facilitate MN loss has not yet been fully identified, but a number of potential mechanisms have been reported. The production of NGF (nerve growth factor) (or related species such as NGF-precursor forms (pro-NGF)) in reactive SOD1-G93A (change of alanine to glycine at position 93 of the SOD1 protein) astrocytes can induce death of rat MNs expressing p75-receptor, through a mechanism that involves nitric oxide and peroxynitrite formation [13]. The increased secretion of pro-NGF and subsequent induction of toxic p75 receptor signalling within neighbouring motor neurons is believed to be a key element of the toxicity of mutant SOD1-expressing astrocytes to motor neurons [14]. Studies in mouse have similarly shown that astrocytes expressing mutant forms of SOD1-G37R (change of arginine to glycine at position 37), SOD1-G85R (change of arginine to glycine at position 85), and SOD1-G93A (change of alanine to glycine at position 93), release soluble factors that can selectively kill embryonic stem (ES) cell-derived or primary MNs *in vitro*, and have demonstrated that neurotoxicity by *SOD1*-*G93A* astrocytes is mediated through the recruitment of the Bax-dependent death machinery. By contrast, conditioned medium from wild-type SOD1-expressing astrocytes displays a supportive/survival effect on MNs similar to that observed with non-transgenic astrocytes [15]. Co-culture of ES cell-derived MNs with *SOD1*-*G93A* astrocytes markedly decreases MN survival relative to primary wild-type *SOD1* astrocytes [16]. *In vivo*, the transplantation of glial-restricted precursor cells into the vicinity of cervical spinal cord respiratory MNs in a *SOD1*-*G93A* rat model of motor neuron disease limits progression of the disease, resulting in enhanced motor and respiratory physiological functions and enhanced survival [17]. The neuroprotective effects have been partly attributed to increased expression of the astrocytic glutamate transporter, GLT1. Taken together, the evidence from these studies suggests that astrocytes are critically involved in MN depletion in ALS, most likely acting through multiple mechanisms.

As yet, no study has examined whether astrocytes exert an effect on MN-progenitor cells. Two factors suggest this is an important question. First, the efficient derivation of mature (Hb9+) MNs from embryonic stem cells is a sought-after goal in the understanding, and potential treatment, of motor neuron diseases: factors that enhance early steps in MN differentiation will therefore contribute to the derivation of MNs *ex vivo*. Second, the realisation that new neurons can be generated within the adult brain suggests the possibility that ALS-driven MN depletion may result from the loss of adult-born neurons. Recent experiments in songbirds show that adult neurogenesis can result in the addition of new neurons to cortical premotor circuits that govern peripheral muscle control [18], while dopamine produced by projections of the brain promotes adult MN regeneration in lesioned spinal cord in zebrafish [19], supporting this idea. Thus, we aimed to extend current knowledge by examining the potential effect of astrocytes on MN progenitors. Here we show that wild-type astrocyte-conditioned medium promotes the efficient generation of MN progenitors from mouse ES cells. By contrast, *SOD1*-*G93A* astrocytes are less supportive for the generation of these MN progenitors.

## Results

### Spatio-temporal expression profiles of transcription factors in MN development in mouse neural tube

As a basis for monitoring MN differentiation from mouse ES cells *in vitro*, we first examined the expression profiles of the transcription factors Olig2, Hb9/MNR2 and Islet1 in the embryonic mouse neural tube at E10.5; all have been previously demonstrated to be involved in MN fate determination [20-22].

**Figure 1:**
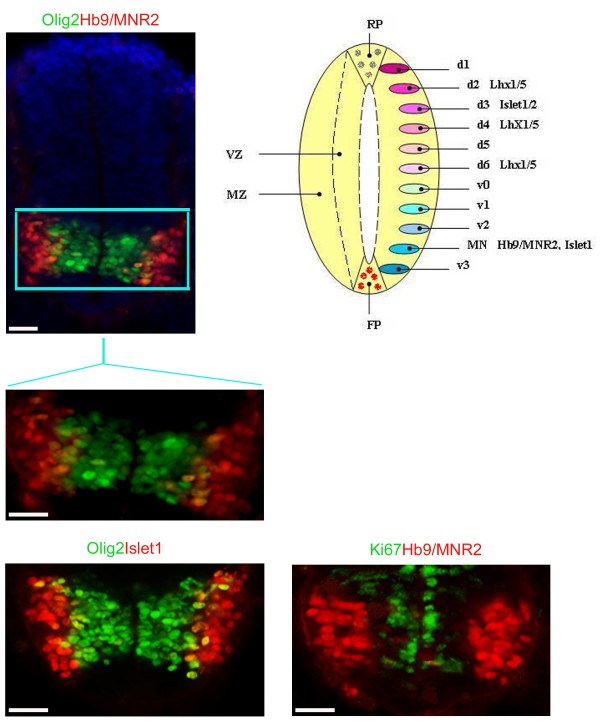
**Motor neuron development in embryonic mouse neural tube.** Fluorescence images of embryonic (E10.5) mouse neural tube, after immunolabelling for Olig2 (green), Islet1 (red), Hb9/MNR2 (red), Ki67 (green) and DAPI for nuclei (blue). Scale bars 50 μm. The schematic of the neural tube shows the dorsal (d1-d6) and ventral domains (v0-v3, MN) of the neural tube and corresponding expression markers. RP: roof plate, FP: floor plate, VZ: ventricular zone, MZ: mantle zone, d: dorsal, v: ventral, MN: motor neuron. Olig2+ cells are restricted to the VZ, the region of proliferating progenitor cells in the developing neural tube. Islet1+ and Hb9/MNR2+ cells are found at the peripheral mantle zone of the neural tube representing post-mitotic neurons. A small number of Olig2+/Hb9+ cells, or Olig2+/Islet1+ cells represent progenitor cells that are making the transition towards Hb9/MNR2+ neurons as they migrate away from the medial VZ to the peripheral MZ.

As previously shown [20-22], Olig2 expression is restricted to the ventral neural tube, demarcating progenitor domain (p)V3 and (p)MN (Figure 1). By contrast, Hb9/MNR2 expression is restricted to differentiated MNs in the mantle zone of the emerging spinal cord (Figure 1). Consistent with this, double labelling of Hb9/MNR2 with Ki67, a marker of proliferating cells, reveals no overlap, confirming that Hb9/MNR2 is a marker for post-mitotic MNs (Figure 1). Double-labelling for Olig2 and Hb9/MNR2 reveals that the majority of Olig2+ cells do not co-express Hb9/MNR2 and are largely restricted to the ventricular zone and subventricular zone (VZ/SVZ), the region of the developing neural tube that harbours proliferating progenitor cells. A small proportion of Olig2+ cells (8.47%) were Hb9/MNR2+. These Olig2+/Hb9+ cells were found furthest from the VZ/SVZ, and represent a population of Olig2+ cells that are making their transition towards Hb9/MNR2+ differentiating MNs as they migrate away from the medial VZ to the peripheral mantle zone (Figure 1). Similarly, a few Olig2+/Islet1+ cells were located away from the VZ/SVZ and were detected immediately adjacent to Olig2-/Islet1+ differentiated MNs (Figure 1). Taken together, our data confirm previous reports [23,24], showing that many Olig2+ cells are proliferating MN progenitor cells. These analyses suggest, further, that the combinatorial analysis of Olig2, Hb9/MNR2 and Islet1 expression can be used as a basis for monitoring the differentiation status of MNs from mouse ES cells.

### Wild-type but not mutant SOD1-G93A astrocyte-conditioned medium promotes the generation of MN progenitors from mouse ES cells and subsequent MN differentiation

**Figure 2:**
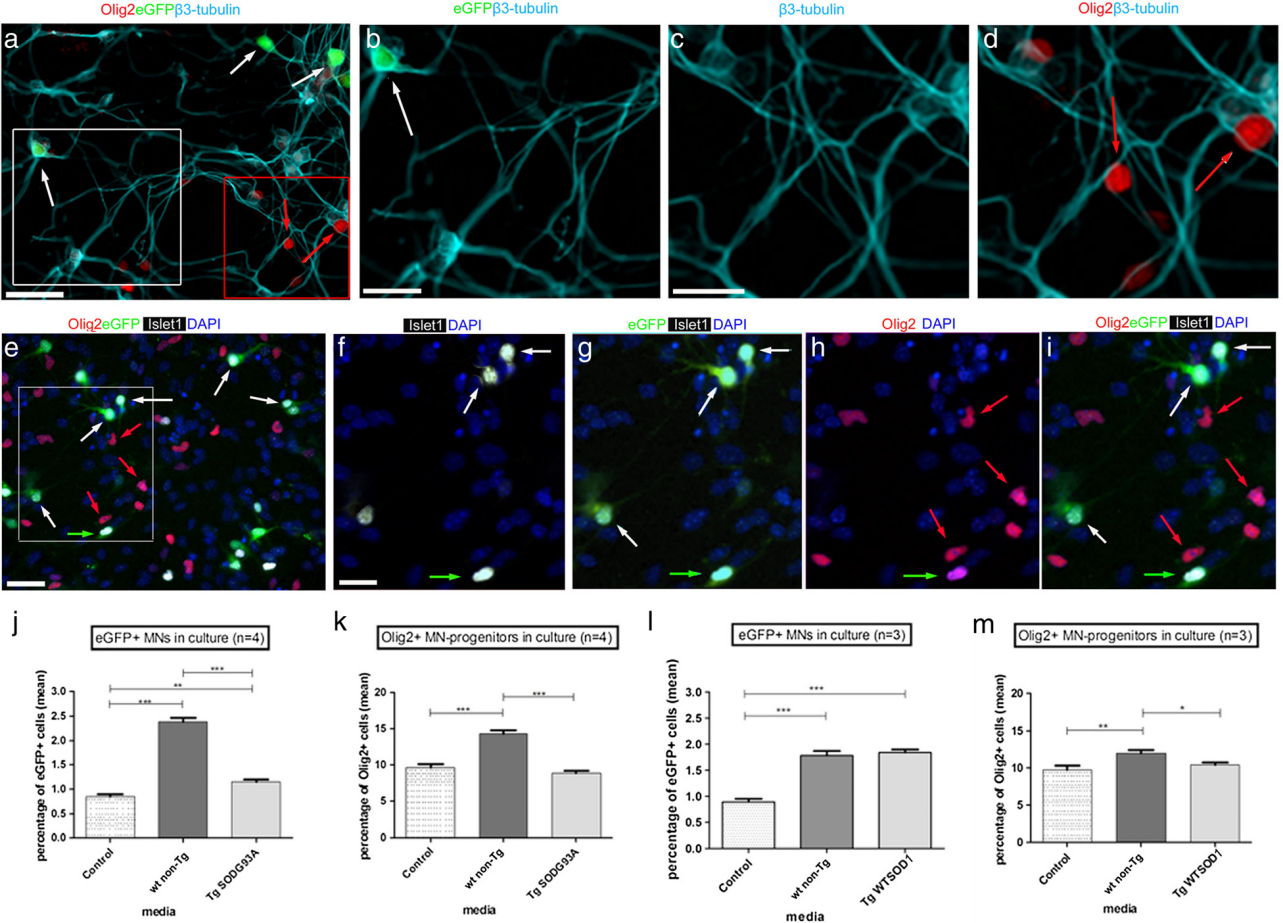
**The effect of astrocyte conditioned media on motor neuron-progenitors and motor neurons derived from differentiation of mouse embryonic stem cells. (a**-**i)** Cultures grown in wt non-Tg astrocyte CM. **(a)** Immunolabelling for Olig2 (red), Hb9:eGFP (green) and β3-tubulin (light blue) showing Hb9:eGFP+β3-tubulin+ cells (white arrows) and Olig2+ cells (red arrows). Scale bar 50 μm. **(b)** High-magnification of white boxed area in **(a)**, showing co-expression of Hb9:eGFP and β3-tubulin (white arrow). Scale bar 33 μm. **(c**, **d)** High-magnification channel views of red boxed area in **(a)**. Olig2+ cells (red arrows) do not co-express β3-tubulin. Scale bar 20 μm. **(e)** Immunolabelling for Olig2 (red), Hb9:eGFP (green), Islet1 (white) and nuclei counterstained with DAPI (blue), showing Hb9:eGFP+Islet1+ cells (white arrows), Olig2+ cells (red arrows) and Olig2+Hb9:eGFP+Islet1+ cell (green arrow). Scale bar 50 μm. **(f**-**i)** High-magnification channel views of white boxed area in **(e)**. MNs show co-expression of Islet1 and Hb9:eGFP (white arrows, **f,g**). Most Olig2+ progenitors do not co-express Islet1 or Hb9:eGFP (red arrows, **i**). Very rarely, an Olig2+Hb9:GFP+Islet1+ cell is detected (green arrow, **f-i**). Scale bar 25 μm. **(j)** A higher proportion of MNs (percentage of total cells) differentiated in wt non-Tg versus control or Tg SOD1G93A media (P<0.001) and, in Tg SOD1G93A CM versus control (P<0.01). **(k)** A higher proportion of MN-progenitors differentiated in wt non-Tg versus control or Tg SOD1G93A media (P<0.001). Comparable proportions of MN-progenitors differentiated in control and Tg SOD1G93A media. **(l)** Comparable proportions of MNs differentiated in wt non-Tg and Tg WTSOD1 CM and these were significantly higher versus control (P<0.001). **(m)** Slightly more MN-progenitors differentiated in wt non-Tg versus Tg WTSOD1 CM (P<0.05). Comparable proportions of MN-progenitors differentiated in control and Tg WTSOD1 CM. Values represent means ± s.e.m from *n=4* or *n=3* independent experiments (indicated in graphs), performed in triplicate, analysed by one-way ANOVA followed by Tukey’s post-test. *P<0.05, **P<0.01, ***P<0.001.

The effect of conditioned medium (CM) from cultured wild-type non-transgenic (wt non-Tg) astrocytes on differentiated MNs was first assessed, in comparison to control medium (not conditioned by astrocytes). MN differentiation was assessed through Hb9:eGFP reporter activity and further confirmed by co-expression of either β3-tubulin or Islet1. Hb9:eGFP+ MNs co-expressed β3-tubulin (Figure 2a,b; white arrows: 100% co-expression, data not shown) and Islet1 (Figure 2e-g; white arrows: 95.03% co-expression, data not shown). Quantitative analyses showed that culture with wt non-Tg astrocyte CM resulted in a marked increase in MNs. The percentage of eGFP+ MNs was increased by a fold-change of 2.8, compared to the control (p <0.001) (Figure 2j), consistent with previous findings that wt non-Tg astrocyte CM results in a highly significant increase in differentiated MNs [15].

To determine whether the increased proportion of differentiated MNs might be due, in part, to enhanced numbers of progenitors, we analysed expression of the b-HLH (basic helix-loop helix) transcription factor, Olig2 [20,25]. Many Olig2+ cells were detected within the cultures (Figure 2a,e). High magnification views after combinatorial labelling with Olig2, Hb9:eGFP and β3-tubulin, or Olig2, Hb9:eGFP and Islet1 revealed that the vast majority of Olig2+ cells did not co-express either β3-tubulin (Figure 2c,d, red arrows) or Islet 1 (Figure 2h,i, red arrows). Quantitative analyses revealed that only a very minor proportion of Olig2+ cells co-expressed β3-tubulin (0.2%) or Islet1 (0.17%) (green arrow, Figure 2e-i). A significantly higher percentage of Olig2+ MN-progenitor cells was detected in cultures grown in wt non-Tg astrocyte CM, compared to those in control medium (Figure 2k). Quantitative analyses showed that the percentage of Olig2+ MN-progenitors in wt non-Tg astrocyte CM was increased by 1.5-fold compared to the control (p <0.001). These data suggest that a primary effect of wt non-Tg astrocyte CM is to increase the number of MN progenitor cells. Importantly, moreover, culture with wt non-Tg astrocyte CM appeared to result in a more robust conversion of Olig2+ progenitors to MNs or survival of MNs. In the case of controls, approximately 8.8% of Olig2+ progenitors differentiated to MNs, whereas with wt non-Tg astrocyte CM, approximately 16.8% of Olig2+ progenitors differentiated to MNs. Thus, wt non-Tg astrocyte CM may promote the generation of differentiated MNs in two ways: first, by increasing the numbers of progenitors, and second, by supporting their robust differentiation/survival.

In contrast to the effects of wt non-Tg astrocyte CM, medium conditioned by astrocytes derived from transgenic mice expressing mutant SOD1-G93A (Tg *SOD1*-*G93A*) did not show a supportive effect on Olig2+ MN progenitors. Quantitative analyses showed that in Tg *SOD1*-*G93A* CM, the number of Olig2+ MN-progenitors was similar to those in the control (p >0.05, not significant), but decreased significantly (p<0.001: a 1.6-fold decrease) compared to the littermate wt non-Tg CM (Figure 2k). Tg *SOD1*-*G93A* astrocyte CM similarly appeared far less able to support eGFP+ MNs, compared to wt non-Tg astrocyte CM. Quantitative analyses revealed a two-fold difference in the percentage of eGFP+ MNs in Tg *SOD1*-*G93A* CM compared to the littermate wt non-Tg CM (p <0.001) (Figure 2j). Notably, however, the efficiency of differentiation to MNs from Olig2+ progenitors in Tg *SOD1*-*G93A* CM appears comparable to that in wt non-Tg astrocyte CM (differentiation/survival coefficient of 13% which is closer to the wt non-Tg astrocyte CM than to the control: see Methods). To determine whether Tg *SOD1*-*G93A* CM decreases the numbers of MN progenitors and MNs by increasing apoptosis, indicative of the presence of a toxic factor [15], we analysed chromatin condensation and nuclear fragmentation after DAPI-labelling [26]. Quantitative analyses revealed no statistically significant association of the number of apoptotic cells with any of the three media (Additional file 1: Figure S1). Taken together, these results indicate that, while CM from wt non-Tg astrocytes is strongly supportive of both Olig2+ MN progenitor cells and eGFP+ MNs, CM from Tg *SOD1*-*G93A* astrocytes appears to lack, in particular, a trophic support factor to motor neuron progenitors.

In order to confirm that the significant differences found in the proportion of Olig2+ MN-progenitor cells and eGFP+ MNs between the wt non-Tg astrocyte CM and the *SOD1*-*G93A* or the control medium were indeed due to the presence of the *G93A* mutation and not to the presence of the transgene, a similar study was performed, this time using conditioned medium from transgenic astrocytes over-expressing human wild-type SOD1 (Tg *WTSOD1*), or, for comparison, CM from their wt non-Tg littermates. As expected, the percentage of eGFP+ MNs detected after culture with either wt non-Tg or *WTSOD1* CM was similar (p >0.05, not significant; Figure 2l), while both media evoked a higher proportion of differentiated MNs than control medium (Figure 2l; p <0.001; a two-fold increase of eGFP+ MNs compared to the control; coefficient of differentiation is 11% for control; 16% for wt non-Tg; 18% for Tg *WTSOD1*). Similarly, there was a significant difference in the percentage of Olig2+ MN-progenitor cells in cultures grown in wt non-Tg astrocyte CM compared to control medium (p <0.01; Figure 2m). However, there was almost no difference in the proportion of Olig2+ cells supported through wt non-Tg murine astrocyte CM, compared to CM from Tg *WTSOD1* littermates (Figure 2m; p <0.05). The similar proportion of Olig2+ progenitors and eGFP+ MNs generated in the presence of wt non-Tg and Tg *WTSOD1* astrocyte CM (Figure 2l,m) suggests that the presence and over-expression of the human transgene in mouse astrocytes is not the reason for any observed differences in the numbers of eGFP+ MNs. Therefore, the difference in the proportion of eGFP+ MNs in the wt non-Tg and Tg *SOD1*-*G93A* astrocyte CM is likely to be due to the effect of the *SOD1*-*G93A* mutation.

### Neither wild type astrocyte CM nor mutant SOD1G93A astrocyte CM affects the generation of Lhx1/5+ interneurons from mouse ES cells

**Figure 3:**
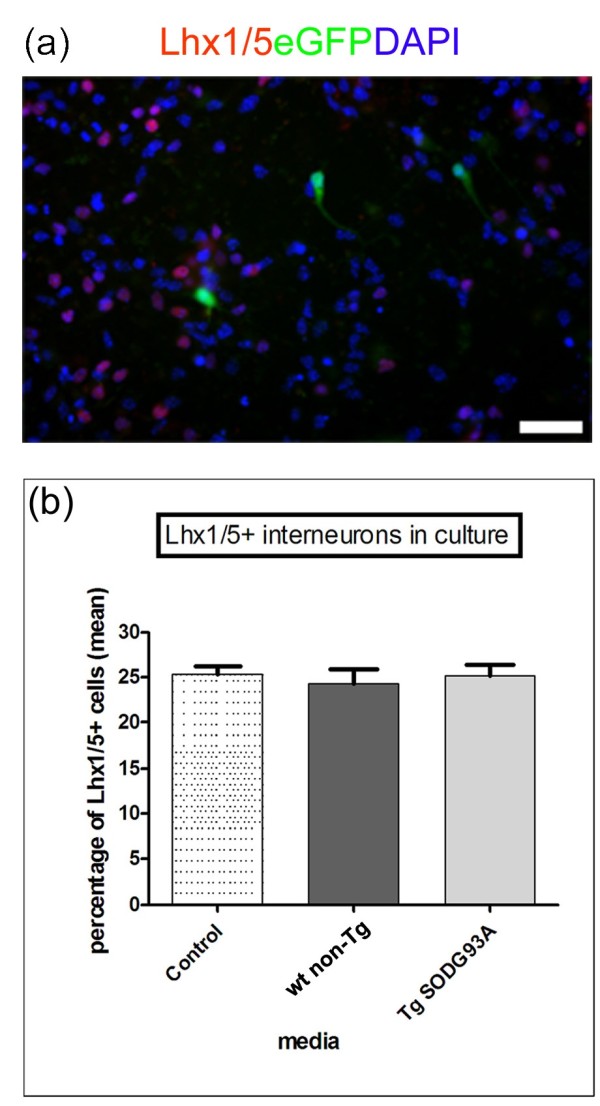
**Examination of the effect of astrocyte conditioned media on interneurons derived from differentiation of mouse embryonic stem cells. (a)** Double-labelled fluorescence image of culture grown in wt non-Tg astrocyte CM labelled for Lhx1/5 interneurons (red), Hb9:eGFP MNs (green) and DAPI for nuclei (blue). Scale bar 50 μm. **(b)** Analysis of the number of interneurons in control medium, wt non-Tg and Tg SOD1G93A CM. Data gathered from 2 independent experiments, each condition examined in triplicate and analysed by one-way ANOVA followed by Tukey’s post-test. No significant differences in the proportion of interneurons were found between the three media.

We next examined whether the observed effects of Tg *SOD1*-*G93A* and wt non-Tg astrocyte CM were specific to MN-progenitors and MNs or whether similar effects might be observed with other types of neurons. Analysis of Lhx1/5+/Hb9- interneurons (Lhx1/5 is the mammalian orthologue of chick Lim1/2, recognised by the anti-Lim1/2 antibody) [27,28] revealed that the proportion of interneurons that differentiated in cultures was very similar for all three types of media examined. Thus, Tg *SOD1*-*G93A* CM, littermate wt non-Tg astrocyte CM and control medium appeared to be equally supportive to interneurons (p >0.05; Figure 3a,b). This comparative study suggests that the supportive factors in wt non-Tg and Tg *WTSOD1* astrocyte CM that are lacking in *SOD1*-*G93A* astrocyte CM may act only on MN progenitor cells and therefore particularly affect differentiated MNs.

## Discussion

Published studies over the last few years have demonstrated negative effects of mutant *SOD1* astrocytes on MN survival [12-14,17] but no study has so far examined any potential effects on MN progenitor cells. The question of how astrocyte CM may impact on MN progenitors is important, both from the perspective of *ex vivo* differentiation strategies and, from the perspective of adult neurogenesis, since buffering of the niche from localised astrocytes may affect survival and differentiation efficiency of the progenitors. Here we demonstrate an impact of astrocyte CM on both MN progenitors and MNs, and demonstrate that mutant *SOD1* astrocytes fail, particularly, to provide survival/trophic support to MN progenitors.

Our study reveals markedly higher numbers of Olig2+ MN progenitor cells and eGFP+ MNs in wt non-Tg astrocyte CM and consistently lower numbers in littermate Tg *SOD1*-*G93A* CM. In contrast, Tg *WTSOD1* and littermate wt non-Tg CM have similar effects on eGFP+ MNs and Olig2+ MN-progenitors, indicating that our findings are likely due to the presence of mutant *SOD1*-*G93A*, rather than over-expressed SOD1 *per se*.

The significantly higher number of Olig2+ MN progenitor cells in the wt non-Tg astrocyte CM supports the idea that normal astrocytes favour MN development potentially by secreting supportive and/or trophic factors that promote MN progenitors. The positive role of astrocytes in promoting MN growth and maturation in *in vitro* cultures is already established [29]. Wild-type and mutant *SOD1* astrocytes produce substantial amounts of functionally-variable factors that can positively or negatively affect MN survival. Such factors include trophic factors (GDNF -glial-derived neurotrophic factor, NGF) [30,31], pro-inflammatory/inflammatory proteins (iNOS -inducible nitric oxide synthase, cystatin-C, interferon-α, prostaglandin-D2) [32,33], proteases (complement 3α,β) and protease inhibitors, carrier proteins (apolipoprotein E, IBP-2 -insulin-like growth factor binding protein-2), extracellular matrix binding proteins (cyclophilin-A) and anti-oxidant proteins (peroxiredoxins) [34]. Our study extends current knowledge by showing that astrocytes also positively affect MN progenitor cells, potentially governing Olig2+ cell-proliferation or Olig2+ cell-survival.

Unlike wt non-Tg astrocyte CM, Tg *SOD1*-*G93A* astrocyte CM does not favour MN progenitor cell growth: similar numbers of Olig2+ cells are found in Tg *SOD1*-*G93A* CM and in control non-conditioned medium. The relatively high numbers of differentiated MNs found with *SOD1*-*G93A* CM, compared to control non-conditioned medium, however, suggests that the mutant astrocyte CM does support MN differentiation/survival. It is therefore likely that a number of the aforementioned factors that are produced by astrocytes and that affect MN survival are secreted in the conditioned medium and are also able to affect MN progenitor cell survival, growth or differentiation.

Our findings support a previous study that shows a halving in the number of primary and ES cell-derived MNs when cultured in Tg *SOD1*-*G93A* astrocyte CM compared to wt non-Tg astrocyte CM [15]. Our results differ from this study, however, in that they provide no evidence for MN death through increased apoptosis of MN progenitors or MNs: nuclear fragmentation was not increased by *SOD1*-*G93A* CM, nor do we observe a significant decrease in Olig2+ MN progenitor cells or MNs in Tg *SOD1*-*G93A* astrocyte CM compared to the baseline level in the presence of control non-conditioned medium. Why might our findings differ from this study, that demonstrated neurotoxicity by *SOD1*-*G93A* astrocytes mediated through the recruitment of the Bax-dependent death machinery? Notably, in our study, we analysed the effects of *SOD1*-*G93A* CM at an earlier time-point. Potentially, then, the neurotoxic effects of *SOD1*-*G93A* CM are realised later than the effects we examine here. While we cannot exclude the possibility that cell-death (through non-apoptotic pathways) contributes to the reduced numbers of MN progenitor cells, our data indicate that the mutant *SOD1*-*G93A* in astrocytes affects a secretory mechanism/product which renders the CM less supportive/trophic to MN-progenitors, potentially affecting either Olig2+ cell-proliferation or Olig2+ cell-survival. Thus, the beneficial effect observed with wt non-Tg CM is likely due to the presence of a supportive factor(s) that is absent or reduced from the Tg *SOD1*-*G93A* CM.

We also determined whether the effects of Tg *SOD1*-*G93A* and wt non-Tg astrocyte CM were specific to MNs and their progenitor cells by examining the effect on Lhx1/5+/Hb9- interneurons. Both the Tg *SOD1*-*G93A* and wt non-Tg CM were as trophic/supportive to interneurons as the control medium indicating that mutant *SOD1*-*G93A* in astrocytes did not affect interneurons and that the effects of wt non-Tg and Tg *SOD1*-*G93A* astrocyte CM were selective for MNs and MN progenitor cells. This is in agreement with Nagai et al. who reported no effects on primary spinal GABAergic or dorsal root ganglion neurons and ES cell-derived interneurons grown in *SOD1*-*G93A* compared to non-Tg astrocyte CM [15].

## Conclusions

Astrocytes are actively implicated in the neurodegeneration in ALS. The exact mechanisms by which mutant *SOD1* in astrocytes can exert negative effects on MNs and MN-progenitors still remain obscure, although existing evidence indicates that astrocytes exert their effects on MNs through several different mechanisms. Our study extends current knowledge by showing a negative effect of the *SOD1*-*G93A* mutation on Olig2+ MN-progenitor cells, possibly revealing new mechanisms of MN loss at the early stages of MN development.

## Methods

### Antibodies

Primary antibodies used were: anti-Olig2 rabbit polyclonal (Chemicon), anti-Lim1/2 (4F2) mouse monoclonal (Developmental Studies Hybridoma Bank), anti-Islet1 (4D5) mouse monoclonal (Developmental Studies Hybridoma Bank), anti-Hb9/MNR2 (5C10) mouse monoclonal (Developmental Studies Hybridoma Bank), β3-tubulin (class 3 beta-tubulin) mouse monoclonal or rabbit polyclonal (Covance), anti-Ki67 rabbit polyclonal (Novocastra). Secondary antibodies used were anti-rabbit IgG-Alexa 594 and anti-mouse IgG-Alexa 594 (Molecular Probes), anti-mouse IgG-Cy5 (Jackson laboratories).

### Animal ethics statement

All experiments were conducted according to the Animal (Scientific Procedures) Act 1986, under Project License 40/3089 reviewed and approved by the University of Sheffield Ethical Review Sub-Committee, and the UK Animal Procedures Committee (London, UK). The UK Home Office code of practice for the housing and care of animals used in scientific procedures was followed. We adhere to the ARRIVE guidelines for reporting animal research [35].

### Immunohistochemistry

Mouse embryos at E10.5 were washed with PBS and fixed in 4% PFA for 2 hours at 4°C, then rinsed with PBS and incubated in 0.2M phosphate buffer with 30% sucrose at 4°C overnight. Whole mount embryos were sectioned in slices of 15 μm thickness using a cryostat at -25°C. Slices were collected on glass slides, allowed to dry for 30 min-1 hour, then washed with PBS, permeabilised with PBS/1% goat serum/0.1% Triton X-100 and incubated with primary antibodies at 4°C overnight. Incubation with secondary antibodies was performed at room temperature for 1 hour. The specimens were mounted in Vectashield Mounting medium with DAPI and sealed with cover glass. Microscopy was performed using ZEISS Axio Imager.Z1 microscope and AxioVision 4.6.3 software.

### Immunocytochemistry

Cell cultures were fixed with 4% paraformaldehyde, permeabilised with PBS/ 2% goat serum/ 0.2% Triton X-100 and incubated with primary antibodies at 4°C overnight. Incubation with secondary antibodies was performed at room temperature for 1 hour and the specimens were mounted in Vectashield Mounting medium with DAPI. A ZEISS Axio Imager.Z1 microscope was used and images were captured using AxioVision 4.6.3 software. Islet1+, β3-tubulin+ and Olig2+ cells were counted in 44 random fields and co-expression of the markers was estimated as a percentage of Olig2+ cells. For apoptosis assessment of Olig2+ and Hb9:eGFP+ cells, apoptotic nuclei exhibiting chromatin condensation and nuclear fragmentation after DAPI-labelling were counted in a total of 30 random fields per condition. For the conditioned media experiments, Olig2+, Hb9:GFP+ and Lhx1/5+ cells (the mammalian orthologue of chick Lim1/2, recognised by the anti-Lim1/2 antibody) were counted in 30 random fields per technical replica of each of the conditions (control, wt non-Tg or Tg) and per experiment and calculated as the percentage of total cells (DAPI nuclei) per field. The percentages calculated from similar conditions within each experiment were pooled together. The mean percentages of positive cells for each marker and per condition were statistically analysed.

### Statistical analysis

The data used for the statistical analyses were collected from n independent biological replicates as noted in the figure legends. In each biological replica, there were 3 technical replicas per condition (control medium, transgenic or wild-type non-transgenic mouse). Ten random fields per technical replica were used for cell counting. Thus at least 30 random fields were used per condition and per experiment; therefore at least 90 random fields were counted in total. Each field contains between 250–300 cells. Therefore for each experiment, a sample size of at least 7500 cells was used per condition. Statistical analysis was performed using GraphPad Prism 5.0 software. Data were examined and fitted a normal distribution and therefore were analysed using one-way ANOVA followed by Tukey’s multiple comparison post-test, with Confidence Interval 95% and alpha=0.05. Differences were considered significant for P values less than 0.05. For apoptosis assessment, association of the number of apoptotic cells with the media was examined using the Chi-square test with 95% Confidence Interval.

### Calculation of differentiation/survival coefficient

For each condition (control, wt non-Tg or Tg conditioned medium) the number of Olig2+ and eGFP+ cells was used in the following formula:

Differentiation/survival coefficient= Y/X *100; where X is the mean percentage of Olig2+ cells of the treatment group; Y is the mean percentage of eGFP+ cells of the treatment group.

The formula translates as follows: In a total of 100 cells, X cells are Olig2+ and Y cells differentiate to eGFP+ cells. Therefore, in 100 Olig2+ cells the proportion of cells to differentiate/survive towards eGFP+ cells is Y/X *100.

### Preparation of SHH-N protein

SHH-N protein containing culture supernatant was prepared as described previously [36]. Briefly, 3 days post transfection of a ShhN-pIRES2-eGFP construct into HEK/293T cells, the culture supernatant was collected and concentrated up to 10× or 25× by repetitive centrifugation in columns of a 10 kDa threshold (Amicon Bioseparations) according to the manufacturer’s instructions.

### Preparation of murine primary astrocyte cultures

Primary astrocyte cultures were prepared from the cortices of *SOD1*-*G93A* (B6SJLTg(SOD1*G93A)1Gur/J purchased from Jackson Laboratories), *WT SOD1* and non-Tg littermate neonate 1–2 day old (p1-p2) mice. The total number of mice used were, eight Tg *SOD1*-*G93A* and ten wt non-Tg mice from four litters, six Tg *WTSOD1* and six wt non-Tg from 3 litters. The cortices were stripped of meninges, washed and triturated in Hank’s balanced salt solution (HBSS) with Ca2+/Mg2+ containing 0.04% trypsin (Sigma), 0.1 mg/ml collagenase (Calbiochem) and 0.05 mg/ml DNaseI (Sigma). Following trituration, the enzymatic process was terminated by addition of an equal volume of complete medium [Dulbecco’s modified Eagle’s medium (DMEM, Cambrex), 10% heat inactivated fetal calf serum (FCS, BioSera), 100 units/ml penicillin and 100 μg/ml streptomycin (Gibco)] and the cells plated in complete medium [10% FCS, 2 mM L-glutamine, 100 U/ml Penicillin and 100 μg/ml Streptomycin] in a T25 (P1). The cell preparations were allowed to attach. After 48 hours, the medium with any cell debris was removed, cells were washed once with PBS and fresh complete medium was added. Astrocytes and microglia were allowed to grow to confluency for 2 weeks.

### Purification of astrocytes from microglia

The overlying microglia cells were removed by shaking overnight on a rotatory shaker (Weiss Gallenkamp) at 37°C between 180-220 rpm. Pure astrocyte cultures were next established by mild trypsinisation (0.25% trypsin and 1mM EDTA in HBSS (-Ca/Mg), added in a ratio of 1:2 with serum-free DMEM/F12), followed by trypsinisation with 2.5% trypsin in PBS and trituration up to single cells. Trypsin was inactivated with complete medium and the suspension was centrifuged. The astrocytes were plated in complete medium in T25 flasks (P2) and allowed to grow to confluency for 10 days before harvesting for conditioned medium.

### Preparation of astrocyte conditioned medium

Conditioned medium was prepared by incubation with the astrocytes. The maintenance medium was removed from the cultures and astrocytes were rinsed with PBS. N2B27 medium (see below) was added and the medium was conditioned for 48 hours. Conditioned medium was collected, centrifuged to remove any cells present and stored at 4°C to be used immediately.

### Differentiation of mouse ES cells in monolayer culture

MNs expressing eGFP were derived from the transgenic Hb9:eGFP mouse ES cell-line HBG3 [37] (generous gift from Professor Thomas Jessell, Columbia University). Mouse ES cells were removed from the flask by trypsinisation, resuspended in mESC-medium [DMEM (4.5 g/L glucose and 0.11 g/L sodium pyruvate), L-glutamine (2 mM), penicillin/streptomycin (100 μg/ml) (all from Gibco), 15% FCS (Globepharm) and 0.1 mM β-mercaptoethanol (Sigma)] and plated on tissue culture plastic substrate pre-coated with 0.1% w/v gelatin overnight. Mouse ES cells-medium was next replaced by N2B27 (1:1) medium. N2 consisted of DMEM:F12, BSA fraction-V (50 μg/ml, Gibco), insulin (25 μg/ml), transferrin (100 μg/ml), progesterone (20 nM), putrescine (100 μM), sodium selenite (20 nM) (all from Sigma), L-glutamine (2 mM), penicillin/streptomycin (100 μg/ml). B27 consisted of neurobasal medium (Gibco), B27 (1:50), L-glutamine (2 mM), penicillin/streptomycin (100 μg/ml). The differentiating cells were cultured in N2B27 for 7 days, RA (0.1 μM) and SHH-N (1×) added on days 3 and 4. On day 7, the cells were trypsinised and replated on PDL (10 μg/ml)/laminin (5 μg/ml) coated 8-well chamber slides at 5×10^4^ cells/cm^2^ in control N2B27 or astrocyte conditioned N2B27 supplemented with bFGF (10 ng/ml) for 2 days. After 2 days, bFGF was withdrawn and fresh control or conditioned N2B27 were added. The differentiation cultures were grown for another 3 days, prior to immunocytochemical analysis.

## Abbreviations

ALS: Amyotrophic lateral sclerosis; bHLH: basic Helix-Loop-Helix; CM: Conditioned medium; ES cells: Embryonic stem cells; MN: Motor neuron; NGF: Nerve growth factor; non-Tg: non-transgenic; SOD1: Superoxide dismutase-1; Tg: Transgenic; SVZ: Sub-ventricular zone; VZ: Ventricular zone; Wt: Wild-type.

## Competing interests

The authors declare that they have no competing interests.

## Authors’ contributions

YAC designed and performed the experiments, performed the statistical analyses, interpreted the results and contributed to the manuscript preparation. KO and MP contributed to the design of the experiments, interpretation of results and manuscript preparation. PJS was the grant holder. PNM, MP and PJS supervised the study and contributed to the manuscript preparation. All authors read and approved the final manuscript.

## Supplementary Material

Additional file 1: Figure S1 Examination of the effect of astrocyte conditioned media on the apoptosis of motor neuron-progenitor cells and motor neurons. (a, b) The apoptotic nuclei of Olig2+ MN-progenitor cells (a) and Hb9:eGFP+ MNs (b) were counted in three independent experiments in a total of 30 random fields per condition. Statistically significant association of the number of apoptotic cells with the media was examined using Chi-square test with 95% Confidence Interval (alpha=0.05). There is no statistically significant association between the level of apoptosis of Olig2+ cells (P value 0.2787) or eGFP+ MNs (P value 0.3028) with any of the three media. Apoptotic Olig2+ cells are 3.98%, 3.22% and 4.51% of the total Olig2+ cells in Tg SOD1G93A CM, wt non-Tg CM and control medium respectively. Apoptotic eGFP+ MNs are 6.32%, 3.11% and 3.82% of the total eGFP+ MNs in Tg SOD1G93A CM, wt non-Tg CM and control medium respectively. Click here for file
