# Supplementary figures and images for: Wild-type but not mutant SOD1 transgenic astrocytes promote the efficient generation of motor neuron progenitors from mouse embryonic stem cells
Source: BMC Neurosci. 2013 Oct 17;14:126. doi: 10.1186/1471-2202-14-126 (PMC3853012; doi:10.1186/1471-2202-14-126)

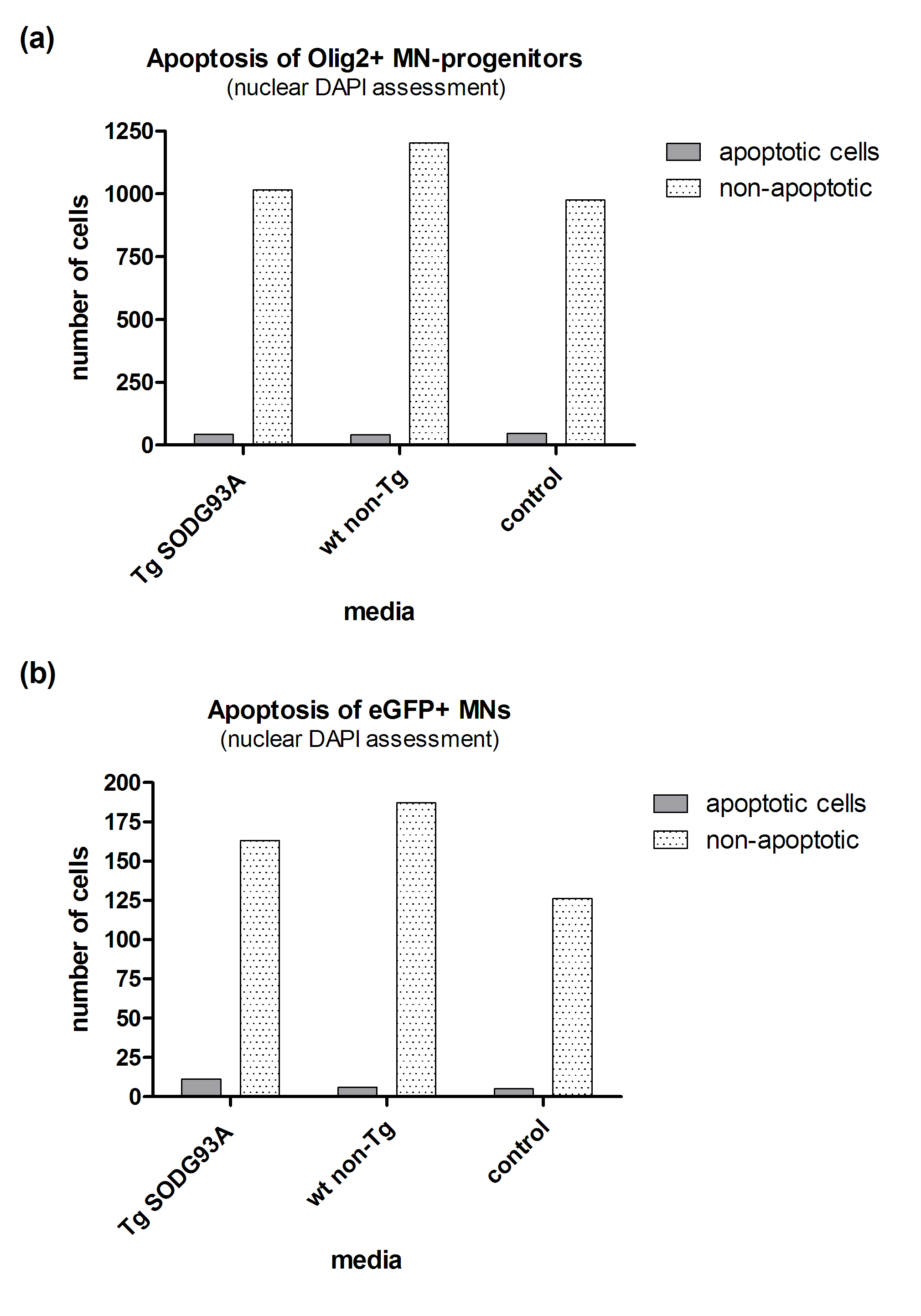

Supplement: Additional file 1: Figure S1 — Examination of the effect of astrocyte conditioned media on the apoptosis of motor neuron-progenitor cells and motor neurons. (a, b) The apoptotic nuclei of Olig2+ MN-progenitor cells (a) and Hb9:eGFP+ MNs (b) were counted in three independent experiments in a total of 30 random fields per condition. Statistically significant association of the number of apoptotic cells with the media was examined using Chi-square test with 95% Confidence Interval (alpha=0.05). There is no statistically significant association between the level of apoptosis of Olig2+ cells (P value 0.2787) or eGFP+ MNs (P value 0.3028) with any of the three media. Apoptotic Olig2+ cells are 3.98%, 3.22% and 4.51% of the total Olig2+ cells in Tg SOD1G93A CM, wt non-Tg CM and control medium respectively. Apoptotic eGFP+ MNs are 6.32%, 3.11% and 3.82% of the total eGFP+ MNs in Tg SOD1G93A CM, wt non-Tg CM and control medium respectively. [file 1471-2202-14-126-S1.jpeg]
